# Supplementary material for: Structural insights into the mechanism of archaellar rotational switching
Source: Nat Commun. 2022 May 23;13:2857. doi: 10.1038/s41467-022-30358-9 (PMC9126983; doi:10.1038/s41467-022-30358-9)
Supplement: Supplementary file 1 — Supplementary Information [file 41467_2022_30358_MOESM1_ESM.pdf]

Supplementary Information for:

Structural insights into the mechanism of archaellar rotational switching

Florian Altegoer[1,2#], Tessa E. F. Quax[3,4], Paul Weiland[1], Phillip Nußbaum[3], Pietro I. Giammarinaro[1], Megha Patro[3], Zhengqun Li[3], Dieter Oesterhelt[5], Martin Grininger[6], Sonja-Verena Albers[3], Gert Bange[1,7#]

[1] Philipps-University Marburg, Center for Synthetic Microbiology (SYNMIKRO) & Faculty of Chemistry, Karl-von-Frisch-Straße 14, 35043, Marburg, Germany
[2] current address: Heinrich-Heine University Düsseldorf, Institute of Microbiology, Universitätsstraße 1, 40225 Düsseldorf, Germany
[3] Archaeal virus-host interactions, Faculty for Biology, University of Freiburg, Schaenzlestrasse 1, 79104 Freiburg, Germany
[4] Biology of Archaea and Viruses, Groningen Biomolecular Sciences and Biotechnology Institute, University of Groningen, Nijenborgh 7
9747 AG Groningen, The Netherlands
[5] Max-Planck Institute for Biochemistry, Am Klopferspitz 18, 82152 Martinsried
[6] Goethe University Frankfurt, Institute of Organic Chemistry and Chemical Biology and Buchmann Institute for Molecular Life Sciences, Max-von-Laue-Straße 15, 60438 Frankfurt am Main, Germany
[7] Max-Planck Institute for terrestrial Microbiology, Karl-von-Frisch-Straße 10, 35043 Marburg, Germany

[#]To whom correspondence should be addressed: altegoer@hhu.de, gert.bange@synmikro.uni-marburg.de

Supplementary information

Table S1. Crystallographic data collection and refinement statistics

	*Mj*CheF	*Ph*CheF	*Mm*CheY:CheF
**Data collection**			
Space group	$P2_12_12$	$P2_1$	$P2_1$
Cell dimensions			
a, *b*, *c* (Å)	169.058 86.144 166.523	50.62 188.6 58.29	56.29 70.87 59.82
α, β, γ (°)	90 90 90	90 112.904 90	90 91.603 90
Resolution (Å)	47.16 - 3.59 (3.719 - 3.591)	46.63 - 2.75 (2.848 - 2.75)	44.07 - 2.3 (2.382 - 2.3)
R_{merge}	0.2395 (1.739)	0.141 (2.366)	0.1551 (1.825)
$I / \sigma I$	10.50 (1.54)	23.1 (1.04)	7.66 (0.88)
Completeness (%)	99.53 (97.16)	99.92 (99.84)	99.61 (99.52)
Redundancy	13.1 (13.9)	13.6 (13.3)	6.5 (5.9)
$CC_{1/2}$	0.998 (0.62)	0.999 (0.536)	0.997 (0.577)
**Refinement**			
Resolution (Å)		46.63 - 2.90	44.07 – 2.30
No. reflections		22251 (2207)	20984 (2090)
R_{work} / R_{free}		23.7/28.4	24.9/30.7
No. atoms			
Protein		5544	3281
Ligand/ion		0	10
Water		0	83
B-factors			
Protein		102.23	80.08
Ligand/ion		-	62.20
Water		-	69.42
Ramachandran (%)			
favored		96.15	96.62
allowed		3.85	3.38
outliers		0.00	0.00
R.m.s. deviations			
Bond lengths (Å)		0.006	0.005
Bond angles (°)		1.03	0.88

*Values in parentheses are for highest-resolution shell.

Table S2. Plasmids used in this study.

Plasmid	***Usage***	***Citation***
pET24d	Protein overexpression	(Novagen)
pGAT2	Protein overexpression with N-terminal GST-tag	(Novagen)
pET24d-CheY_m. mari	Plasmid for expression of N-terminal his-tagged CheY protein of *M. maripaludis* S2 in *E. coli*. pET24d (Novagen®) backbone.	[1]
pGAT2-MmCheF	Plasmid for expression of GST-CheF of *M. maripaludis* S2 in *E. coli*	(this study)
pGAT2-MmCheF$_{CTD}$	Plasmid for expression of GST-CheFΔN of *M. maripaludis* S2 in *E. coli*	(this study)
pGAT2- MmCheF$_{NTD}$	Plasmid for expression of GST-CheFΔCTD of *M. maripaludis* S2 in *E. coli*	(this study)
pGAT2- MmCheF$_{\Delta\alpha 8}$	Plasmid for expression of GST-CheFΔα8 of *M. maripaludis* S2 in *E. coli*	(this study)
pGAT2- MmCheF$_{\alpha 8}$	Plasmid for expression of GST-CheFα8 of *M. maripaludis* S2 in *E. coli*	(this study)
pET24d-MmCheY:CheF$_{CTD}$	Plasmid for expression of CheY:CheF$_{CTD}$ of *M. maripaludis* S2 in *E. coli*	(this study)
pET24d-MmCheF	Plasmid for expression of N-terminal his-tagged CheF protein of *M. maripaludis* in *E. coli*. pET24d (Novagen®) backbone.	(this study)
pET24d-TkCheF	Plasmid for expression of N-terminal his-tagged CheF protein of *T. kodakarensis* in *E. coli*. pET24d (Novagen®) backbone.	(this study)
pET24d-PhCheF	Plasmid for expression of N-terminal his-tagged CheF protein of *P. horikoshii* in *E. coli*. pET24d (Novagen®) backbone.	(this study)
pTA1228	Protein expression plasmid for *H.volcanii*. Amp resistance and pyrE2 marker. Tryptophan inducible.	[2]
pIDJL-40	Protein expression plasmid to use in *H.volcanii* for C-terminal GFP tagging based on pTA1228.	[3]
pSVA3922	Protein expression plasmid to use in *H.volcanii* for N-terminal GFP tagging based on pTA1228.	[4]
pSVA5078	pIDJL-40 with *H. volcanii* cheF1. Epression plasmid to express CheF-GFP in H.vol	(this study)
pSVA5079	pSVA3922 with *H. volcanii* cheF1. Expression plasmid to express GFP-CheF1 under trp promoter.	[4]
pSVA5657	Based on pSVA5079. Expression plasmid to express an N-terminal-GFP fused CheF1 truncation (truncated 83 aa at the C-terminus) in *H.volcanii* GFP-CheF1ΔC (Δ83aa)	(this study)
pSVA5658	Based on pSVA5079. Expression plasmid to express an N-terminal-GFP fused CheF1 truncation (truncated 14 aa at the C-terminus) in *H.volcanii* GFP-CheF1Δα8 (Δ14aa)	(this study)

Table S3. Primers used in this study.

Number	*Name*	*Sequence*	*Description*	*Reference*
7233	CheF1_NdeI_fw	GGAATTCCATATGAAACCCGGTGAGCAAAAGCTCGGGG	forward primer for amplification of CheF1 of H.volcanii for cloning in pIDJL-40 to create pSVA5078	(this study)
7234	CheF1_BamHI-STOP-rev	CGGGATCCCTGCTCGTTGATGGCGTCCG	reverse primer for amplification of CheF1 from H.volcanii for cloning in pIDJL_40 to create pSVA5078	(this study)
11342	HvcheF1ΔC (Δ83aa)_Fw	GCCGTCAGTTTCCTCTAGGGATCCACTAGTTCTAGAGCGG	forward primer to create a 83 aa C-terminal truncation of CheF of H.volcanii to clone into pSVA5079 to create pSVA5657	(this study)
11343	HvcheF1ΔC (Δ83aa)_Rev	ACTAGTGGATCCCTAGAGGAAACTGACGGCGCGGTCGGAG	reverse primer to create a 83 aa C-terminal truncation of CheF of H.volcanii to clone into pSVA5079 to create pSVA5657	(this study)
11344	HvcheF1Δα8 (Δ14aa)_Fw	GAGGTGCAACTCAACTAGGGATCCACTAGTTCTAGAGCGG	forward primer to create a 14 aa C-terminal truncation of CheF of H.volcanii to clone into pSVA5079 to create pSVA5658	(this study)
521	MjCheF-BsaI-F	AGGAGGGTCTCccatggGCATAGACAAATCCTCAGAA	Forward primer to create a full-length MjCheF construct	(this study)
522	MjCheF-BsaI-R	AGGAGGGTCTCctcgagCTATTCCACCGTTTC	Reverse primer to create a full-length MjCheF construct	(this study)
523	TkCheF-BsaI-F	AGGAGGGTCTCccatggGCACCATTGCACAGGTT	Forward primer to create a full-lengt TkCheF construct	(this study)
524	TkCheF-BsaI-R	AGGAGGGTCTCctcgagTCACATCACGCCCAT	Reverse primer to create a full-length TkCheF construct	(this study)
530	MmCheF-dN245-BsaI-F	AGGAGGGTCTCCCATGGGCACCATTAAAAGTTTACTTCC	Foward primer to create a $MmCheF_{CTD}$ construct	(this study)
527	MmCheF-dC-BsaI-R	AGGAGGGTCTCCTCGAGTTAGTTGTGATATTTTGTTAATTT	Reverse primer to create a $MmCheF_{NTD}$ construct	(this study)
532	MmCheF-BsaI-F	AGGAGGGTCTCCCATGGGCAGTGCCAAATCTAAA	Forward primer to create MmCheF constructs	(this study)
533	MmCheF-BsaI-R	AGGAGGGTCTCCTCGAGTTAGAAATTTGTAATAATAAAGTTTGTA	Reverse primer to create MmCheF constructs	(this study)
535	MmCheF-a8-F	AGGAGGGTCTCCCATGGGCAGCGGCGAAAAAGGAAGAGCAGTTACAAACTTTATTATTACAAATTTCTAACTCGAGGAGACC	Forward primer to create a MmCheF construct of helix α8	(this study)
536	MmCheF-a8-R	AGGAGGGTCTCCTCGAGTTAGAAATTTGTAATAATAAAGTTTGTAACTGCTCTTCCTTTTTCG	Reverse primer to create a MmCheF construct of helix α8	(this study)

		CCGCTGCCCATGGGAGACC		
603	MmCheY2-BsaI-6H-F	AGGAGGGTCTCccatggGCCATCATCACCATCACCACAGTATTGTAAAAACAATGATTGTAGATGAT	Forward primer to create the CheY fragment for the CheY:$CheF_{CTD}$ fusion construct	(this study)
604	MmCheY2-CheF-BsaI-R	AGGAGGGTCTCCGGAACCAGACCAGCAGACGGACCCTGGAACAGAACGGGAAACAATTTGTTAAACTG	Reverse primer to create the CheY fragment for the CheY:$CheF_{CTD}$ fusion construct	(this study)
605	MmCheF-CheY-BsaI-F	AGGAGGGTCTCGTTCCGCGTGGTTCTGGTGGTATCGAAGGTGGATCCATGGGCACCATT	Forward primer to create the CheF fragment for the CheY:$CheF_{CTD}$ fusion construct	(this study)
606	MmCheF-BsaI-R	AGGAGGGTCTCctcgagTTAGAAATTTGTAATAATAAAGTTTGTAAC	Reverse primer to create the CheF fragment for the CheY:$CheF_{CTD}$ fusion construct	(this study)
619	MmCheF-d8-BsaI-R	AGGAGGGTCTCCTCGAGTTATGTTAAATCTGTTTCTTTCCTAATTCT	Reverse primer to create a MmCheF deletion construct devoid of helix $\alpha 8$	(this study)

Table S4. Strains used in this study.

Name	***Species***	***Background***	***Genotype***	***Used Plasmid***	***Reference***
H26	*H. volcanii*		*ΔpyrE2*		[5]
HTQ403	*H. volcanii*	H26	ΔpyrE2ΔcheF1	pSVA5058	[1]
HTQ96	*H. volcanii*	HTQ403	ΔpyrE2ΔCheF1::pTA1228	pTA1228	[1]
HTQ355	*H. volcanii*	HTQ403	ΔpyrE2ΔcheF1::CheF1-GFP	pSVA5078	(this study)
HTQ356	*H. volcanii*	HTQ403	ΔpyrE2ΔcheF1::GFP-CheF1	pSVA5079	[4]
HTQ577	*H. volcanii*	HTQ403	ΔpyrE2ΔcheF1::GFP-cheF1_ΔC	pSVA5657	(this study)

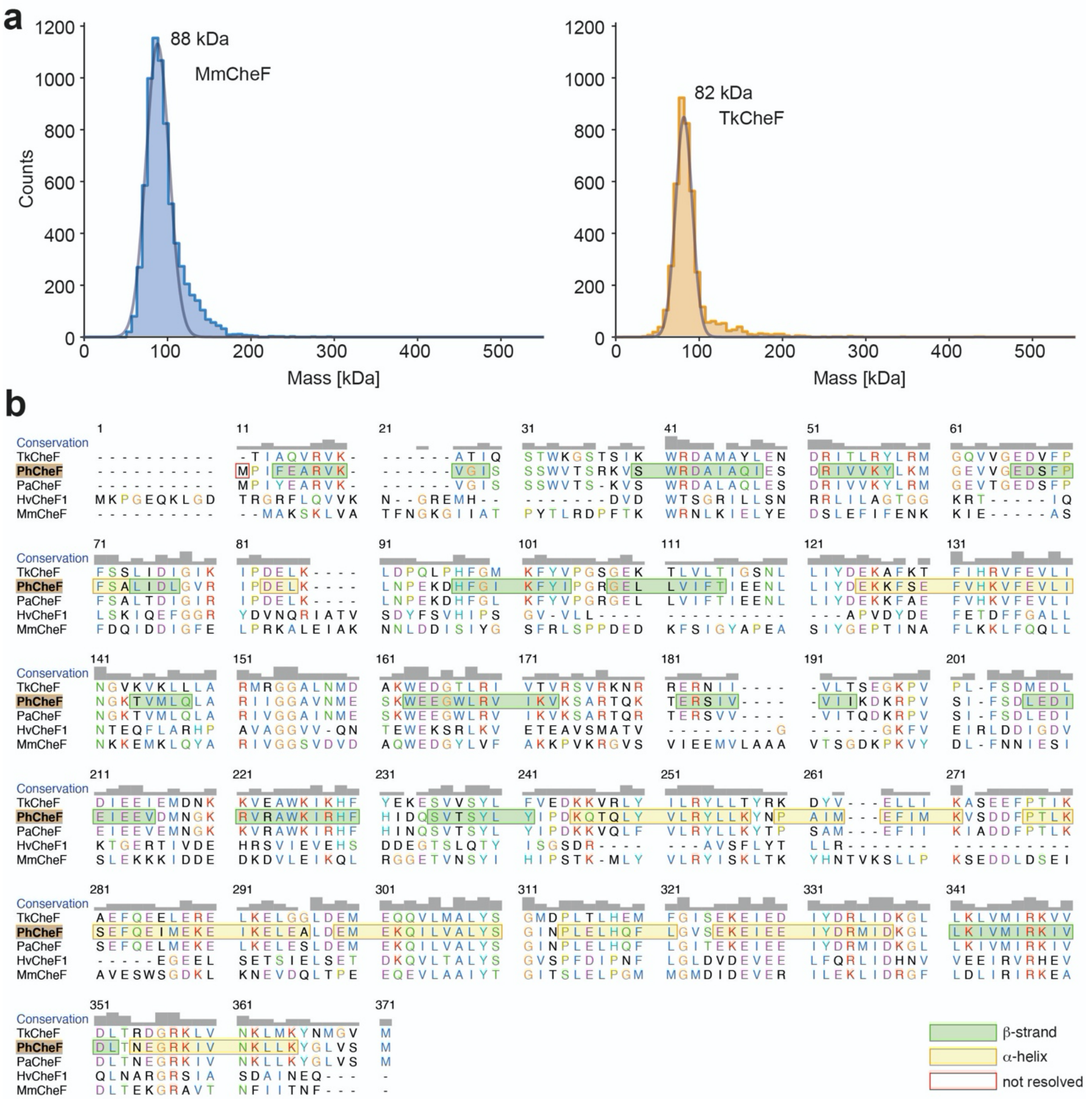

Supplementary Figure 1. a. Mass photometry of CheF from *Methanococcus maripaludis* (Mm, Left) and *Thermococcus kodakarensis* (Tk, right) showing a single species of 88 and 82 kDa, respectively. **b.** Amino acid sequence alignment of CheF's from different archaeal species. Residues are colored according to the Clustal X coloring scheme that depends on the residue type and conservation pattern in the respective column. The actual secondary structure of PhCheF is plotted onto the alignment. The alignment has been generated in Chimera [6]. Source data are provided as a Source Data file.

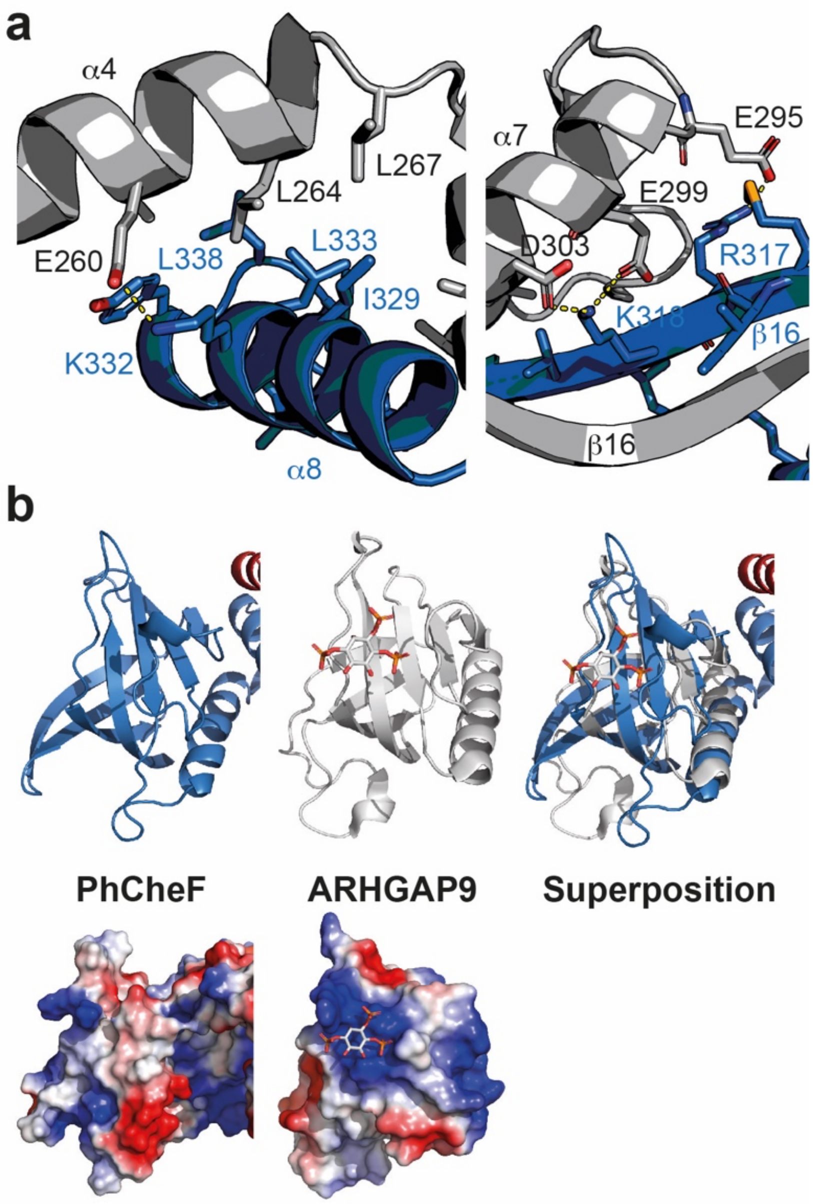

Supplementary Figure 2. a. Interaction interface between the two CheF monomers within the CheF dimer. Salt bridges are indicated with yellow dashed lines. **b.** Structural superposition between the PH domain of CheF and a PH domain of ARHGAP9 (PDB-Code: 2P0F).

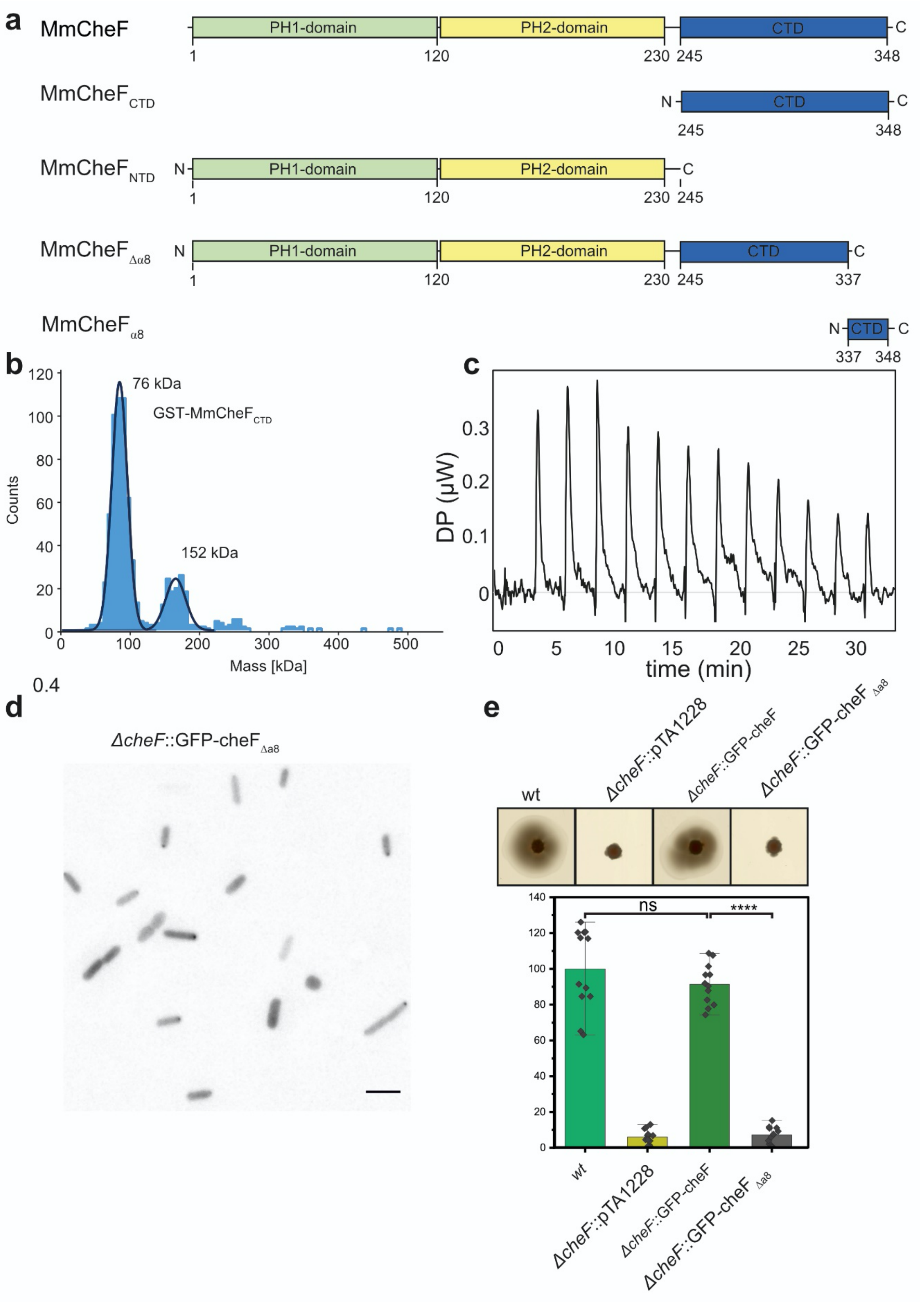

Supplementary Figure 3. a. Schematic drawing of the CheF constructs generated for GST-interaction assays. **b.** Mass photometry of GST-$CheF_{CTD}$ from *Methanococcus maripaludis* shows a dimeric species of 76 kDa, a tetrameric species of 152 kDa and small fractions of higher oligomeric states. **c.** Isothermal titration calorimetry (ITC) of *Mm*CheY and GST-*Mm*$CheF_{\Delta NTD}$ in the absence of BeF_2 and NaF. GST-*Mm*$CheF_{\Delta NTD}$ was added to the sample cell and titrated with *Mm*CheY. **d.** Fluorescent image of Δ*cheF* cells expressing GFP- $CheF_{\Delta\alpha8}$. The scale bar represents 5 µm. **e.** Motility rings of different *H. volcanii* strains on semi-solid

agar plates made of YPC medium. Quantification of the diameter of the motility rings such as shown in a. The experiment was performed with at least 3 technical and 2 biological replicates. WT, *H. volcanii* H26; Δ*cheF*, *H. volcanii* H26 deleted for CheF; pTA1228, empty plasmid; $cheF_{\Delta\alpha 8}$, encoding CheF protein with 8 aa C-terminal truncation. ns, not significant (p=0.251). ****P<0.0001 (p= 0.00000000000000003736) as calculated with unpaired two-sided T-test. Data are represented as mean values +/- standard deviation. n= 13 experiments Source data are provided as a Source Data file.

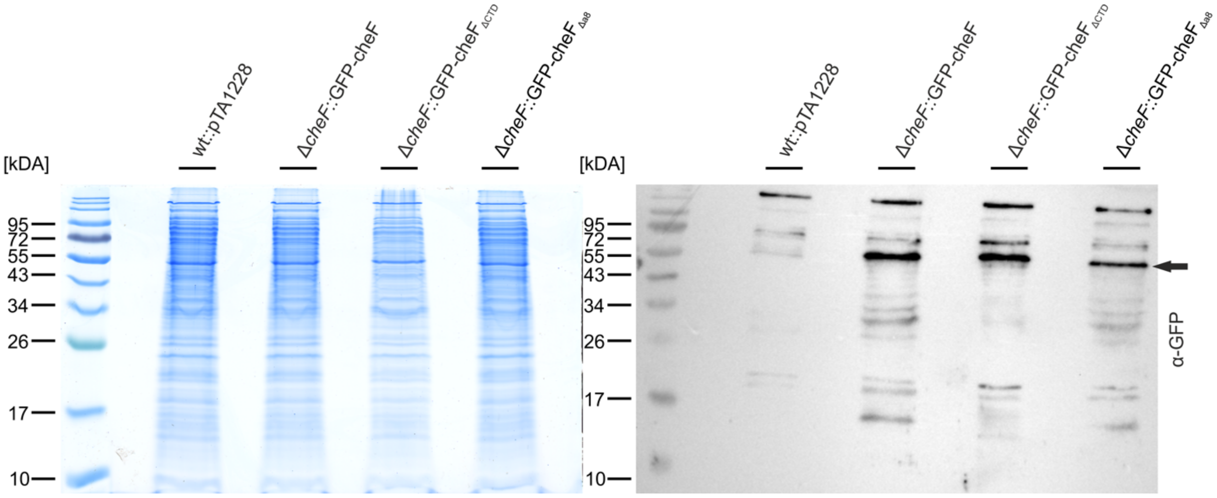

Supplementary Figure 4. Anti-GFP Western-Blots. To confirm the integrity of the GFP-CheF phusion proteins, the expressed constructs were blotted and detected via antibodies against the GFP-tag. Left: loading control of the wildtype harboring an empty expression plasmid, ΔcheF strain expressing either GFP-CheF (59.2 kDA), GFP-CheF$_{\Delta CTD}$ (49.2 kDA) or GFP-CheF$_{\Delta\alpha 8}$ (57.7 kDA). Right: Corresponding Western-Blot. The expressed constructs run at around the same height and are indicated by an arrow. The experiment has been repeated three times independently with similar results. Source data are provided as a Source Data file.

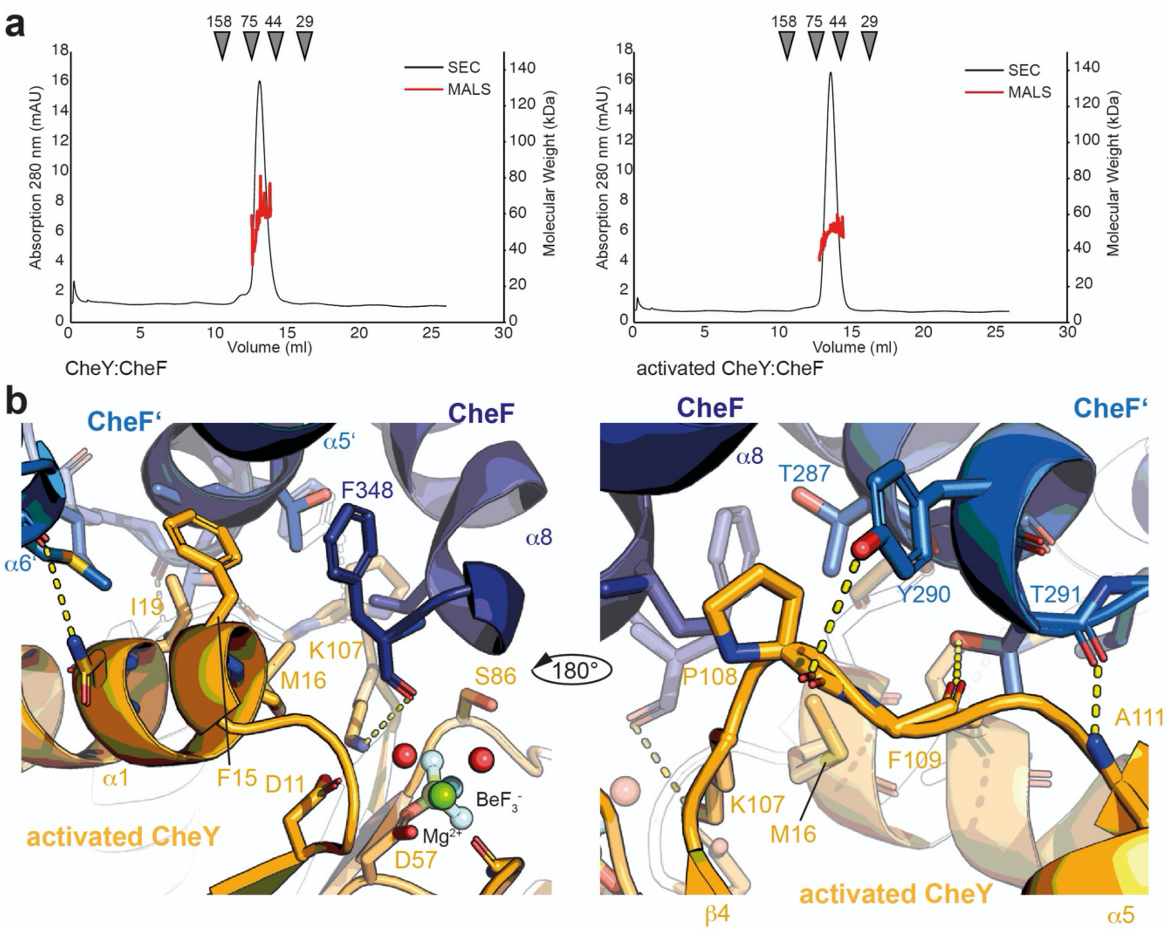

Supplementary Figure 5. a. Multi-angle light scattering (MALS) coupled SEC analysis of MmCheY:CheF$_{CTD}$ in the absence (left) and presence (right) of BeF_2 and NaF. The red curve shows the molecular weight as determined by MALS and the black chromatogram the absorbance at 280 nm. The masses above the chromatogram correspond to the components of the gel filtration calibration kit: Carbonic anhydrase (29 kDa), Ovalbumin (44 kDa), Conalbumin (75 kDa) and Aldolase (158 kDa). Source data are provided as a Source Data file. **b.** Detailed view on the CheY-CheF interface. CheY is colored in orange and CheF molecules are shown in blue and dark blue, respectively. Residues involved in the interaction are displayed as sticks. Electrostatic interactions are depicted as dashed yellow lines.

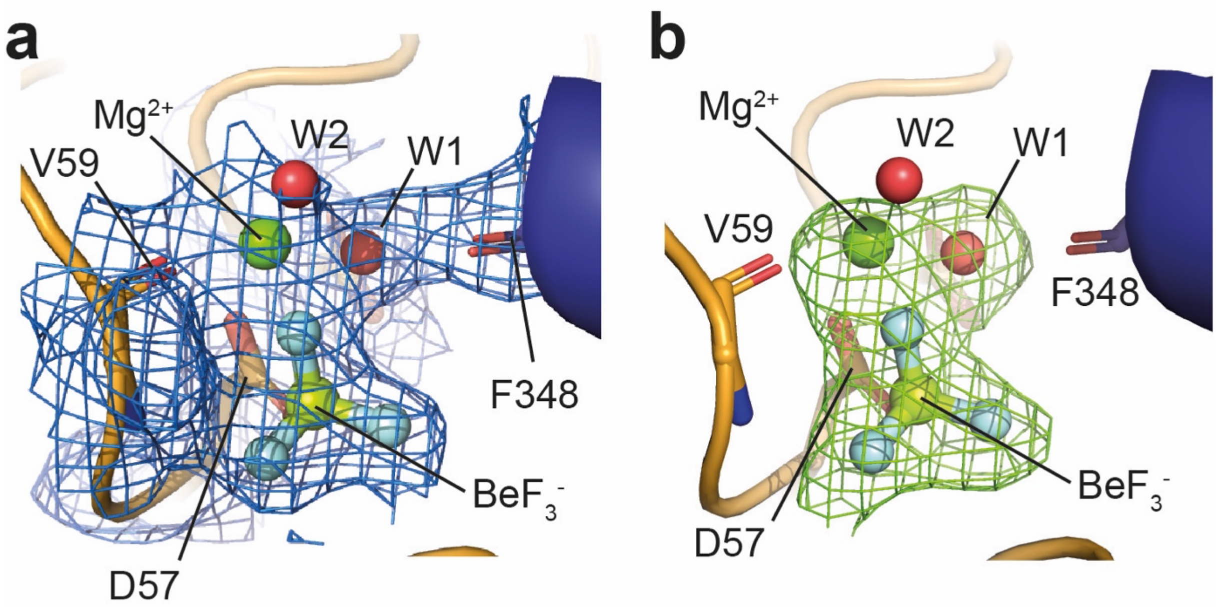

Supplementary Figure 6. Electron density of BeF_3^- and magnesium coordination. a. 2*Fobs-Fcalc* map contoured at 1σ showing the coordinating residues at CheY, waters, magnesium and BeF_3^-. **b.** *Fobs-Fcalc* map contoured at 3σ prior to refinement of waters, magnesium and BeF_3^-.

Supplementary References

1. Quax, T. E. F. *et al.* Structure and function of the archaeal response regulator CheY. *Proc. Natl. Acad. Sci. U. S. A.* **115**, E1259–E1268 (2018).
2. Brendel, J. *et al.* A complex of Cas proteins 5, 6, and 7 is required for the biogenesis and stability of clustered regularly interspaced short palindromic repeats (crispr)-derived rnas (crrnas) in Haloferax volcanii. *J. Biol. Chem.* **289**, 7164–7177 (2014).
3. Duggin, I. G. *et al.* CetZ tubulin-like proteins control archaeal cell shape. *Nature* **519**, 362–365 (2015).
4. Li, Z. *et al.* Positioning of the motility machinery in halophilic archaea. *MBio* **10**, (2019).
5. Allers, T., Ngo, H. P., Mevarech, M. & Lloyd, R. G. Development of additional selectable markers for the halophilic archaeon Haloferax volcanii based on the leuB and trpA genes. *Appl Env. Microbiol* **70**, 943–953 (2004).
6. Pettersen, E. F. *et al.* UCSF Chimera?A visualization system for exploratory research and analysis. *J. Comput. Chem.* **25**, 1605–1612 (2004).
